# Diagnostic performance of a *Strongyloides* IgG4 Rapid Test in detecting human *Strongyloides stercoralis* infection

**DOI:** 10.1186/s13071-025-07154-7

**Published:** 2025-12-11

**Authors:** Rubén Cimino, Ernesto Candela Senti, Tamara García, Sofía Ciotta, Elvia Nieves, Carolina Goizueta, Rahmah Noordin, Nor Suhada Anuar, Victoria Periago

**Affiliations:** 1 Facultad Regional de Orán, Instituto de Investigaciones de Enfermedades Tropicales (IIET), Universidad Nacional de Salta, Alvarado 751, Orán, CP4530 Salta, Argentina; 2 Facultad de Ciencias Naturales, Cátedra de Química Biológica, Universidad Nacional de Salta, Salta, Argentina; 3 Consejo Nacional de Investigaciones Científicas y Técnicas (CONICET), Buenos Aires, Argentina; 4 Facultad de Farmacia y Tecnología Farmacéutica y Parasitología, Universidad de Valencia, Valencia, Spain; 5 Department of Parasitology and Medical Entomology, Faculty of Medicine, Universiti Kebangsaan Malaysia, Kuala Lumpur, Malaysia; 6 Institute for Research in Molecular Medicine, Universiti Sains Malaysia, Penang, Malaysia; 7 Fundación Mundo Sano, Buenos Aires, Argentina

**Keywords:** Soil-transmitted helminths, *Strongyloides stercoralis*, Serology, *Strongyloides* IgG4 rapid test (SsRapid), In-house NIE-ELISA, Argentina

## Abstract

**Background:**

Soil-transmitted helminths (STH) affect more than 1 billion people worldwide. Due to the morbidity in children caused by the accumulation of infections with these parasites, the World Health Organization (WHO) developed deworming programs to reduce worm burden, providing guidelines for *Ascaris lumbricoides*, *Trichuris trichiura*, and hookworms, since they are diagnosed and treated using the same tools. Recently, the WHO has provided guidelines for *Strongyloides stercoralis*, given it requires specific tools for diagnosis and treatment, with the goal to reduce prevalence and encourage the study of new diagnostic algorithms for deworming campaigns in areas where all STHs are present. Herein, we present an evaluation of the SsRapid, a prototype serological test developed to detect *S. stercoralis* IgG4.

**Methods:**

A cross-sectional study was conducted in indigenous communities of Puerto Iguazú (Misiones) during 2023, an endemic area for STH in Argentina. Stool samples were analyzed using coprological methods (sedimentation and Baermann) for helminth parasite detection. Serum samples were analyzed for *S. stercoralis*-specific antibodies using a standardized in-house NIE-ELISA and the SsRapid test. Diagnostic performance was assessed through two analytical frameworks: (i) conventional analysis using coprological methods as a reference standard and (ii) latent class analysis (LAC) to account for the imperfect nature of all diagnostic tests and estimate true sensitivity and specificity without assuming a gold standard.

**Results:**

A total of 327 stools and 226 serum samples were collected and processed. The overall copro-parasitological prevalence of all species of STH, including *S. stercoralis*, was 69.7%. Hookworm was the most prevalent STH detected (59.0%), followed by *S. stercoralis* (25.7%). The seroprevalence of *S. stercoralis* using SsRapid and in-house NIE-ELISA was 51.3% (95% CI 44.8–57.8) and 39.4% (95% CI 33.1–45.8), respectively; a statistically significant difference (*P* = 0.0049) was observed between the assays. Compared to coprological methods, the diagnostic sensitivities of the SsRapid and in-house NIE-ELISA were 86.8% [95% CI 74.6–94.5] and 69.8% [95% CI 55.6–81.6], respectively. LAC, which does not assume a perfect gold standard, estimated a higher true prevalence of 30.7% and identified SsRapid as the most sensitive test (94.3%) and copro-parasitological methods as the most specific (95.9%). The model demonstrated adequate class separation (entropy = 0.68).

**Conclusions:**

Both conventional analysis and latent class modeling consistently demonstrated the superior sensitivity of SsRapid compared to in-house NIE-ELISA and copro-parasitological techniques. The LAC further strengthened these findings by providing unbiased estimates that confirmed SsRapid as the most sensitive test (94.3%) and revealed a higher true disease burden (30.7%) than apparent by coprological methods alone. Therefore, SsRapid is a promising field diagnostic tool for detecting *S. stercoralis* in deworming programs.

**Graphical Abstract:**

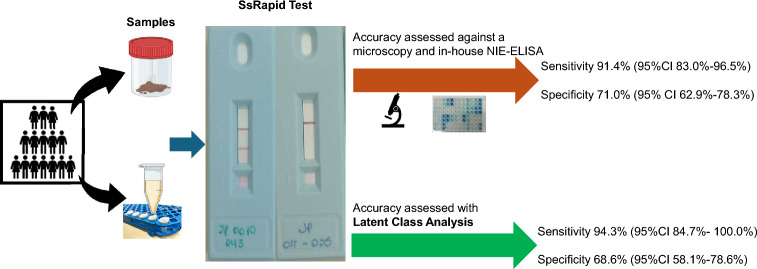

**Supplementary Information:**

The online version contains supplementary material available at 10.1186/s13071-025-07154-7.

## Background

Soil-transmitted helminthiases (STH) are included in the World Health Organization’s (WHO) list of neglected tropical diseases (NTDs) since they are among the most common infections, affecting more than 1 billion people worldwide [1]. The causative agents are nematode parasites transmitted through the fecal-oral route, *Ascaris lumbricoides* and *Trichuris trichiura*, and those transmitted percutaneously, hookworms (*Necator americanus* and *Ancylostoma duodenale*), and *Strongyloides stercoralis*. Due to the morbidity in children caused by the accumulation of infections with these parasites, the WHO developed deworming programs to reduce the worm burden [2]. For strongyloidiasis, the aim is to cure, given the autoinfection cycle and the possibility of reactivation [3]. Since 2001, member states have endorsed the control program through the World Health Assembly resolution (WHA54.19). The main target is to reduce infection by all the STHs, except *S. stercoralis*, in pre-school and school-aged children (PSAC and SAC, respectively) using either albendazole (ALB) or mebendazole (MEB), as measured by coprological and Kato-Katz quantification techniques [2]. Recently, specific guidelines for *S. stercoralis* were published, including prevalence thresholds based on coprological and serological techniques and using ivermectin to treat this helminth infection. Additionally, the target population has been widened to include women of reproductive age and adults with high-risk occupations [4].

One program target is to establish efficient strongyloidiasis control in SAC. To achieve this goal, a diagnostic technique for *S. stercoralis* should be affordable and field-friendly. Ideally, it can be used with the Kato-Katz technique to detect other STHs. The new guidelines establish the use of coprological tests (either the Baermann technique or agar plate culture) or serological tests for detecting *S. stercoralis* infection in either SAC or the entire community [5].

A significant challenge in evaluating new diagnostic tests for strongyloidiasis is the lack of a perfect gold standard. Established coprological methods like the Baermann technique have suboptimal sensitivity due to low and intermittent larval output, while serological tests can cross-react with other helminths. This limitation complicates the accurate assessment of test performance. To address this methodological hurdle, statistical approaches such as latent class analysis (LCA) are increasingly employed. LCA allows for the estimation of true infection status and diagnostic accuracy of multiple tests simultaneously, without relying on an error-free reference, providing a more robust framework for validation in the absence of a definitive standard [6, 7].

Puerto Iguazú in the province of Misiones (Argentina) has a high prevalence of STH, particularly hookworm and *S. stercoralis* [8, 9]. Research conducted in indigenous rural communities adjacent to the urban area of Puerto Iguazú has demonstrated a correlation between environmental factors—including inadequate access to safe water, sanitation, hygiene, and substandard living conditions—and the prevalence of intestinal parasites [9]. Recent research has examined the effectiveness of rapid diagnostic tools, including the SsRapid prototype cassette test, which targets IgG4 antibodies specific to *S. stercoralis* infection. The findings consistently reveal that these assays offer promising diagnostic performance. Nevertheless, the sensitivity and specificity of SsRapid may differ according to regional and population-based factors. This variability shows the need for continual review and adjustment of rapid diagnostic tests to maintain accuracy in different settings [10, 11]. In the current study, we evaluated the use of a rapid test in an area of Argentina highly endemic for *S. stercoralis* and hookworms. Furthermore, we utilized LCA to overcome the limitation of an imperfect gold standard and to provide a statistically robust evaluation of the test’s accuracy compared to conventional parasitological methods [9, 12].

## Methods

### Study area and participants

A cross-sectional study was conducted in the villages of the Mbyá Guaraní ethnic group in Puerto Iguazú, Misiones, Argentina. From April to July of 2023, individuals > 1 year old from the villages of Jasy Porá, Tuba Mbaé, Miri-Marangatú, and Fortín Mbororé were invited to participate in the study by providing stool and blood samples for the detection of intestinal parasites (IPs), with a focus on STHs. This study is part of an ongoing research project; therefore, the study area and sampling methodology have been previously described [9, 12]. Briefly, stool and blood samples were collected during household visits. Sterile containers were distributed and collected on the following day; households were visited up to three times until a single stool sample was returned.

### Stool sample processing and parasitological examination

All fecal samples were analyzed (*n* = 327) using the Telemann sedimentation method and the Baermann method for the detection of *S. stercoralis* and other helminths (*Ascaris*, hookworm, *Trichuris*). Given the high prevalence of hookworm in the study area and the potential for hookworm eggs to hatch and release larvae in fresh, unfixed samples, the microscopic identification of *S. stercoralis* larvae was performed with strict attention to distinguishing morphological characteristics by experienced parasitologists [13, 14]. Samples were processed promptly after collection to minimize the risk of advanced larval development, which could complicate identification. Only larvae displaying the complete set of morphological characteristics consistent with *S. stercoralis* were recorded as positive. Each participant provided a single stool sample. The results of the different coprological techniques were recorded on paper and then transcribed to an Excel spreadsheet. Samples that tested positive for STH were further processed using the Kato-Katz technique [15]. The positive and negative results were used as the reference standard (RS). Detection of all the IPs is presented in the Additional file Table S1 and Additional file Dataset S1.

### Serum

**Fig. 1:**
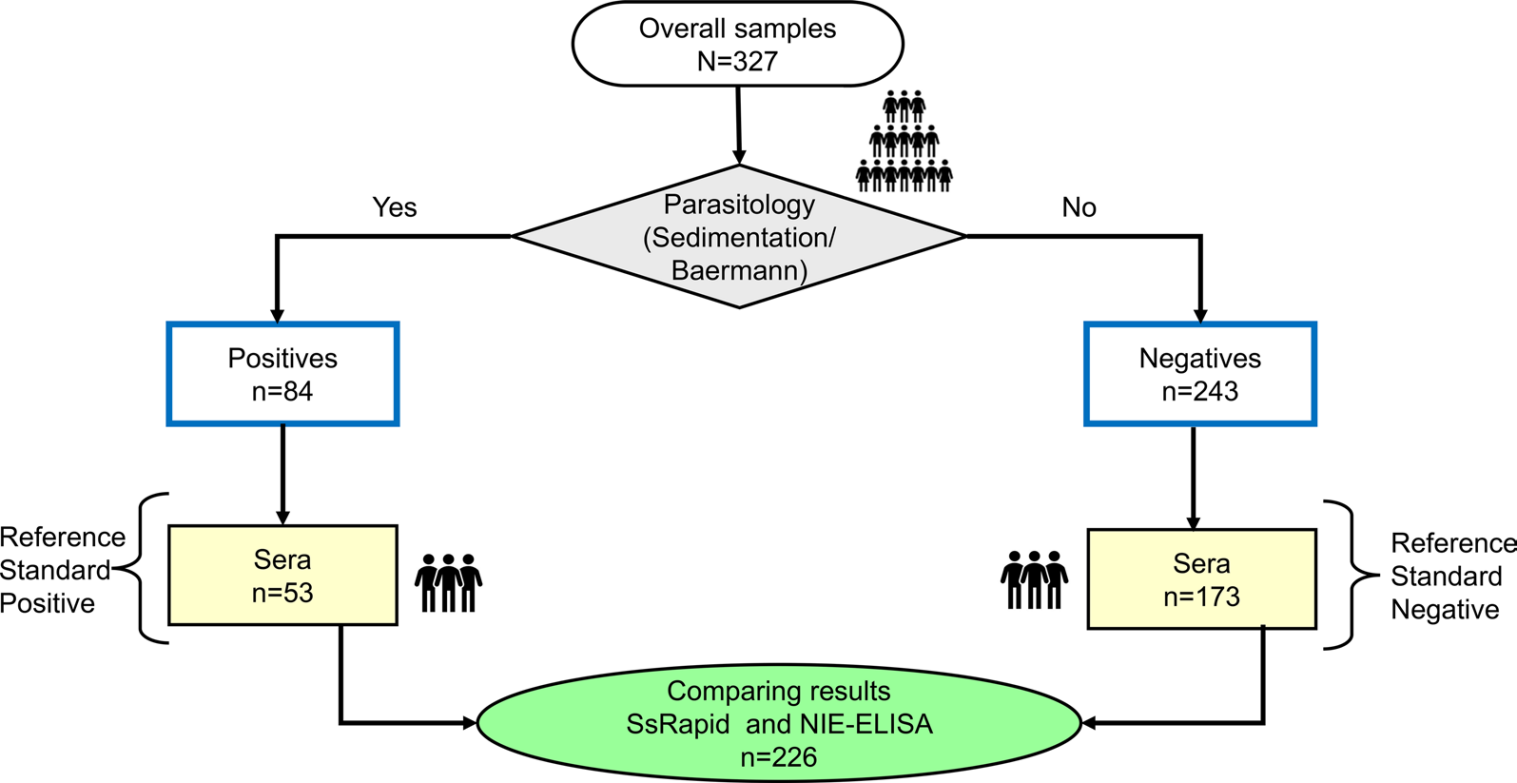
Study flow chart, samples collected, and types of analysis performed

Blood samples (*n* = 226) were collected through venipuncture into serum tubes and transported at room temperature (RT) to the field laboratory and then centrifuged at 1300 × g for 10 min at RT. The supernatant (serum) was aliquoted and kept at − 20 ºC until transported to the analytical laboratory, where the serological tests were performed (Fig. 1).

### SsRapid cassette test

A prototype *Strongyloides* IgG4 rapid test (SsRapid) was used as previously described [16]. Briefly, a serum sample (35 μl) was placed in the sample well and allowed to flow up the nitrocellulose strip by capillary action. *Strongyloides*-specific IgG4 in the serum binds to the recombinant NIE antigen (rNIE) on the test line. The dried gold-conjugated anti-human IgG4 in the oval top well was then reconstituted and added to the test strip. After 15 min from the last step, the result was read as positive if two red lines (test and control lines) were visible on the nitrocellulose strip and negative if only one red (control) line was visible. Positive results were usually observed after 5–10 min. The SsRapid results were interpreted independently by two investigators.

### In-house NIE-ELISA

*Strongyloides stercoralis* infection was detected using an NIE antigen from the laboratory of Dr. T. Nutman (NHI, Bethesda, Maryland, USA) [17]. The enzyme-linked immunosorbent assay with rNIE (NIE-ELISA) was performed as previously described, with some modifications [18]. The micro-well plate was sensitized with 100 μl/well of rNIE antigen at 0.15 μg/ml in coating buffer and incubated at 4 ºC. After antigen sensitization, the plate was washed four times with PBS/0.1% Tween, then blocked for 1 h with mixed nickel chloride 10 mM in PBS/0.1% Tween [19]. Human serum samples were tested at 100 μl/well at 1:100 dilution in PBS Tween/1% skim milk and incubated for 1 h at 37 ºC. Then, human anti-IgG-Fc (Jackson Immuno Research, West Grove, PA) at 1:20.000 dilution was added and incubated for 1 h at 37 ºC. Finally, 3,3’,5,5’-tetramethylbenzidine (TMB) and hydrogen peroxidase (3%) were added (100 μl/well) for 15 min in the dark at RT. The reaction was stopped by adding 100 μl/well 0.5 N H_2_SO_4_. The signal was read at A450 nm using the Bioteck ELx800 instrument (Biotek Instruments, Inc., Winooski, VT, USA).

The cut-off value was determined by constructing a receiver-operating characteristic curve (ROC) and AUC analysis based on ELISA results from participants with parasitological evidence of larvae, considered positive samples (*n* = 18), and individuals without infection, considered negative samples (*n* = 11), from a non-endemic area (the city of Salta). The cut-off value was based on the optimal combination of diagnostic sensitivity and specificity, with an area under the ROC curve of 0.91 (95% CI 0.80–1.00); 80% sensitivity and 95% specificity were obtained.

### Statistical analysis

Only samples with data from three diagnostic methods were considered for analysis (*n* = 226). Various parameters, including sensitivity (Se), specificity (Sp), predictive value, accuracy, and prevalence, were estimated, including their 95% confidence interval (95% CI), using free statistical calculators in MedCalc Software Ltd. [20]. The Se and Sp of each test, NIE-ELISA and SsRapid, were defined as the proportion of positive or negative results over all result samples at the RS. The composite reference standard (CRS) was based on a combination of the parasitological test and NIE-ELISA result (positive/negative). The Se index of SsRapid and in-house NIE-ELISA was compared using the McNemar test. Prevalence was determined by dividing the number of samples that tested positive through copromicroscopy and serological techniques by the total number of samples examined. *P* < 0.05 was considered significant.

Due to the absence of a perfect reference standard for *S. stercoralis* infection, latent class analysis (LCA) was used to estimate the Se and Sp of three diagnostic tests: parasitological (sedimentation/Baermann technique), SsRapid, and in-house NIE-ELISA. LCA is a mixture modeling approach that identifies unobserved (latent) classes based on patterns of responses to observed categorical variables [21]. We specified a two-class model representing the true but unobserved infection status: Class 1 (infected) and Class 2 (non-infected). The model assumes that within each latent class, the three diagnostic tests are conditionally independent. Model parameters were estimated using maximum likelihood estimation. The conditional independence assumption was verified by examining the model residuals. The two-class solution was selected based on theoretical grounds and supported by goodness-of-fit indices, including Akaike information criterion (AIC), Bayesian information criterion (BIC), and entropy. Sensitivities were derived as the probability of a positive test result among individuals in the “infected” latent class, while specificities represented the probability of a negative test result among those in the “non-infected” class. Prevalence was estimated from the class proportions. The analysis was conducted in Jamovi software [22] using the poLCA package through the R interface [23]. A thorough description of the data organization is given in Additional file Dataset S1.

## Results

### Prevalence of intestinal parasites (protozoa and helminths)

The current study involved 327 individuals with a mean age of 16.21 (range, 1–83) years; 68.5% of the study participants were < 15 years old. The sex distribution was 52.3% (*n* = 171) females and 47.7% (*n* = 156) males.

The overall prevalence of IPs was 72.2% (236/327). The most prevalent protozoans were *Blastocystis hominins* 56.9% (186/327) and *Entamoeba coli* 49.2% (161/327). The cumulative prevalence of all STH infections determined by copro-parasitology was 65.4% (214/327). Hookworm was the most common STH infection with an overall prevalence of 59.0% (193/327), followed by *S. stercoralis* with 25.7% (84/327), while *A. lumbricoides* and *T. trichiura* infections were the least prevalent (Additional file 1: Table S1).

### Sensitivity of the serological test compared to copro-parasitology

**Fig. 2:**
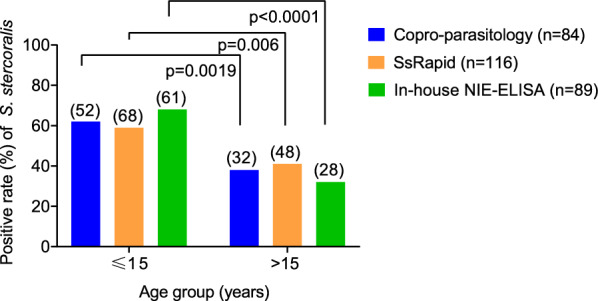
Frequency of samples positive by each of the techniques used in the study conducted in Puerto Iguazú, Misiones, Argentina. Fecal samples processed using copro-parasitology (sedimentation and Baermann) and serum samples processed by the in-house NIE-ELISA and the SsRapid test. The graph shows results separated for participants aged ≤ 15 years and those > 15

To evaluate the serology for the diagnosis of current *S. stercoralis* infection, we investigated the antibody response against rNIE. The seroprevalence was highest using the SsRapid test 51.3% (116/226), while it was 39.4% (89/226) with the in-house NIE-ELISA. The addition of the serological assay approach resulted in an estimated overall prevalence of *S. stercoralis* rising from 25.7% to 50.0% using the SsRapid (Table 1). Among samples positive for *S. stercoralis* by fecal examination, 71.0% also tested positive for hookworm. Age stratification revealed that 50.0% of *S. stercoralis* infections occurred in the younger age group (< 15 years old). Significant differences (*p* < 0.01) in *S. stercoralis* positivity rates were observed between children (≤ 15 years) and adults (> 15 years) across all three diagnostic techniques (Fig. 2).

**Table 1:** Diagnostic parameters of the techniques used in detecting *Strongyloides stercoralis* infection: the SsRapid and NIE-ELISA compared to copro-parasitology methods (sedimentation and Baermann) and the SsRapid compared to a composite reference (copro-parasitology methods and NIE-ELISA)

Diagnostic method	*n*	No. positives	Sero-prevalence (%) [95% CI]	Se. (%) [95% CI]	Sp. (%) [95% CI]	Prev.^1^(%) [95% CI]	PPV (%) [95% CI]	NPV (%) [95% CI]	Accuracy^2^ (%)
SsRapid	226	116	51.3 [44.8–57.8]	86.8 [74.6–94.5]	59.5 [51.8–66.9]	25.7 [21.0–30.4]	42.6 [37.6–47.7]	92.9 [86.6–96.3]	66.5 [59.9–72.6]
In-house NIE-ELISA	226	89	39.4 [33.1–45.8]	69.8 [55.6–81.6]	69.9 [62.5–76.6]		44.5 [37.6–51.7]	87.0 [81.4–91.1]	69.9[63.4–75.8]
SsRapid	Composite (copro-parasitology + in-house NIE-ELISA)								
	226	116	51.3 [44.8—57.8]	91.4 [83.0–96.5]	71.0 [62.9–78.3]	25.7 [21.0–30.4]	52.2 [45.6–58.7]	96.0 [92.1–98.0]	76.2 [70.2–81.7]

^1^Disease prevalence is the number of positives analyzed by copro-parasitology methods/total samples

^2^Overall probabilities that a patient is correctly classified*. Se* sensitivity

*Sp* specificity, *Se* sensitivity, *Prev* prevalence, *PPV* positive predictive value, *NPV* negative predictive value

### Ig4 antibodies of the SsRapid test display high diagnostic accuracy for infection

The diagnostic accuracy of SsRapid was evaluated by constructing 2 × 2 contingency tables based on the RS for both positive and negative cases (Additional file 2: Fig. S1-A). The CRS enabled the positive results of in-house NIE-ELISA to compensate for the lower sensitivity of the copro-parasitological techniques (Additional file: Fig. S1-B). The performance of serological tests (SsRapid and in-house NIE-ELISA) is shown in Table 1. IgG4 displayed the highest sensitivity (Se: 86.8%, 95% CI 74.6–94.5) as well as seroprevalence (51.3%, 95% CI 44.8–57.8) compared to in-house NIE-ELISA. A CRS analysis enhanced both the sensitivity and specificity of the SsRapid test for diagnostic accuracy. A statistically significant difference was found in the detection of *S. stercoralis*-specific antibodies between SsRapid and in-house NIE-ELISA (*P* = 0.0049).

### Individual SsRapid test performance using LAC

Latent class analysis, which demonstrated adequate class separation (entropy = 0.68), revealed distinct and complementary performance profiles for the three diagnostic approaches when a perfect gold standard was not assumed (Table 2). The SsRapid test emerged as the superior screening tool, demonstrating the highest sensitivity (94.3%) for case detection. In contrast, traditional parasitological methods provided the highest specificity (95.9%) for confirmatory diagnosis. The in-house NIE-ELISA occupied an intermediate position, balancing both parameters. The estimated true prevalence of strongyloidiasis in this study was 30.7%, while using the copro-parasitological method, it was 25.7%.

**Table 2:** Diagnostic accuracy of three diagnostic tests for *Strongyloides stercoralis* infection estimated by latent class analysis (sample size = 226, *CI* confidence interval)

Diagnostic method	Sensitivities (%) [95% CI]	Specificities (%) [95% CI]
Parasitology	67.0% [CI 52.4–87.6]	95.90% [90.6–100.0]
SsRapid	94.3% (84.7–100.0)	68.6% [58.1–78.6]
In house NIE-ELISA	79.0% [65.0–93.2]	77.9% [71.0–86.0]
Estimated prevalence	30.7%	

## Discussion

Strongyloidiasis is a public health problem in both endemic and non-endemic countries. The success rate of parasitological diagnosis in detecting larvae in fecal matter is rather poor, and multiple sample collections are required to improve its sensitivity. Thus, a recent WHO report states that evaluating new diagnostic tools is essential for NTD programs [24]. The recombinant NIE antigen of *S. stercoralis* has been identified as a potential biomarker for diagnosing *S. stercoralis* infection. Several reports from different laboratories and regions have indicated that NIE-ELISA’s sensitivity ranged from 70 to 95%, while NIE-LIPS achieved between 97 and 100% sensitivity. The NIE antigen demonstrated specificity with serum samples from patients diagnosed with filariasis, trichuriasis, ascariasis, and hookworm, indicating no observed cross-reaction. The diagnostic specificities among healthy individuals from non-endemic regions ranged from 93 to 100% [14, 17–19].

The lateral flow rapid test (SsRapid) is an alternative diagnostic tool that uses the recombinant NIE antigen of *S. stercoralis* and detects specific IgG4 antibodies. Several other studies have demonstrated its good diagnostic performance [10, 16, 25–28]. Studies conducted in migrant populations from sub-Saharan Africa and SAC in Ecuador have determined their Se and Sp to be between 79–83% and 74–94%, respectively. The rapid test represents an improvement in diagnosis compared to the traditional parasitological and serological methods (ELISA or indirect immunofluorescence) [16, 27, 29]. In Argentina, the in-house NIE-ELISA was used in studies conducted in other areas of the country (northwest localities), whereby the regional *Strongyloides* seroprevalence was approximately 20% [18]. In Puerto Iguazú, Misiones, located in the northeast, reports of IPs, measured by coprological methods, exceeded 50%, with a high prevalence of both *S. stercoralis* and hookworm [9, 12]. Given the good performance of the SsRapid and the in-house NIE-ELISA in other areas in Argentina and other countries, in this study, we tested the performance of the SsRapid at the community level in the indigenous villages of Puerto Iguazú.

Based on fecal examination, this study demonstrated a high detection rate (25.7%) for *S. stercoralis* using coprological methods. Serological tests revealed higher positivity rates, 39.4% for the in-house NIE-ELISA and 51.3% for the SsRapid. Using a combination of sedimentation and Baermann methods as the reference test, the diagnostic sensitivity of the in-house NIE-ELISA and the SsRapid was 69.8% and 86.8%, respectively. When we employed a CRS, SsRapid sensitivity increased to 91.4%.

This study showed that the positivity rate and diagnostic Se of SsRapid were superior to the in-house NIE-ELISA. Various reports have highlighted the advantages of SsRapid over ELISA using native and recombinant antigens. Differences in antigen preparations (recombinant versus native), test formats (ELISA versus lateral flow), and detection antibodies (IgG versus IgG4) may explain the different results obtained by the two serological tests [16].

Our study showed that the prevalence adjusted by previous results using parasitological methods was significantly higher than the calculated prevalence. Thus, the use of parasitological prevalence tends to underestimate the global prevalence. The adjusted parasitological (37.6%) and serological (50.0%) prevalences were significantly higher than those recommended by WHO for initiating a control program (> 10% and > 25%, respectively).

Our study provides insights by comparing traditional gold standard-based evaluation with LCA, which does not assume a perfect reference test. The LCA approach estimated a higher prevalence (30.7% vs 25.7%) and generally higher sensitivity estimates for all tests, suggesting that the conventional parasitological methods used as a gold standard may miss some true infections. Both methods consistently identified SsRAPID as the most sensitive test (86.8–91.4% by traditional methods vs 94.3% by LCA) and parasitological methods as the most specific (95.9% by LCA). The higher sensitivity estimates from LCA likely reflect its ability to account for imperfect reference standards, as parasitological methods are known to have suboptimal sensitivity for strongyloidiasis detection. The LCA approach, while methodologically robust for situations without a perfect gold standard, assumes conditional independence of tests. This assumption may be partially violated in our study since SsRapid and in-house NIE-ELISA both target the same NIE antigen, potentially creating residual correlation. The consistency of performance patterns across both analytical approaches supports the validity of our main conclusions regarding the complementary roles of different diagnostic methods. Furthermore, our results are consistent with those reported by Tamarozzi et al. [7]. In their research, the authors presented the accuracy of the immunochromatographic test (ICT-IgG4), which employs a methodology comparable to that assessed in our investigation (SsRapid). The sensitivity and specificity reported by Tamarozzi et al. [7] for the ICT were 86.3% (95% CI 80.1–92.5%) and 73.9% (95% CI 67.0–80.8%), respectively. Notably, the samples used by Tamarozzi et al. [7] to calculate sensitivity and specificity were from a biobank, and they corresponded to immigrants from sub-Saharan Africa, whereas our study used a cohort from a different population.

The convergence of findings across methods reinforces SsRapid’s role as a good screening test due to its high sensitivity, while parasitological methods remain valuable for specific confirmation. The intermediate characteristics of in-house NIE-ELISA make it a useful adjunctive test, particularly in serial testing algorithms. Our study has several limitations. First, the LAC assumed conditional independence of diagnostic tests, which may not hold perfectly given that SsRapid and NIE-ELISA share the same antigenic target. Second, the sample size, while adequate for LCA estimation, limits the precision of some confidence intervals. Third, the composite reference standard used in traditional analysis, while more comprehensive than single methods, still represents an imperfect gold standard.

The high hookworm prevalence (59%) in our study population raises legitimate concerns about potential cross-reactivity. However, several factors mitigate this concern: (i) the rNIE antigen has demonstrated *S. stercoralis* specificity in previous validation studies [19, 30]; (ii) our LCA-derived strongyloidiasis prevalence (30.7%) is epidemiologically plausible and distinct from hookworm prevalence; (iii) the observed test performance patterns suggest meaningful discrimination between infections; (iv) the detection of IgG4 is deemed useful as a means of increasing the specificity of diagnostic tests for intestinal helminth infections [7]. Nevertheless, some cross-reactivity may contribute to the moderate specificity of SsRapid, warranting caution in hookworm-endemic areas.

Nonetheless, our results suggest the need for community-level intervention through mass drug administration (MDA) in our study area (ivermectin, single dose of 200 μg/kg orally) [5]. The high prevalence of hookworm and *S. stercoralis* detected in Puerto Iguazú aligns with those previously reported [9]. Very few cases of *A. lumbricoides* or *T. trichiura* were detected; these parasites are mainly transmitted via fecal oral routes, and habits such as handwashing and the availability of large water streams for bathing may have contributed to the low prevalence. The study population is highly anemic (> 70%), is malnourished, and has a high prevalence of intestinal parasites (protozoa and STH), further highlighting the need for intervention through MDA [9, 12]. The significant differences in detecting *S. stercoralis* by all methods in children (≤ 15 years old) compared to adults (> 15 years old) were probably due to the much larger proportion (60%) of children among the participants. Furthermore, epidemiological analysis suggests that STH infection occurs at very early ages. The study area has previously been described as hosting a vulnerable population with poor sanitation and hygiene conditions [12]. This study is subject to several important limitations that should be considered when interpreting the findings. First, the sample size was determined based on the assumption that the copro-parasitological test would serve as the gold standard for diagnosis. Therefore, the minimum sample size required for latent class analysis (LCA) was constrained, potentially affecting the robustness of the LCA outcomes. Second, the diagnostic approach in this investigation did not incorporate additional molecular methods such as PCR or quantitative PCR (qPCR). These techniques are recognized for their high sensitivity and specificity and are valuable for confirming cases or excluding potential cross-reactions. The absence of PCR-based diagnostics may limit the ability to definitively identify *S. stercoralis* infections and distinguish them from other parasitic infections. Finally, the study was conducted in an area characterized by a high prevalence of hookworm infection. This epidemiological context presents challenges in obtaining accurate estimates of the specificity of the SsRapid test. The presence of hookworm at high frequencies may introduce confounding factors, making it difficult to fully assess the test’s ability to discriminate between *S. stercoralis* and other helminth infections.

## Conclusions

The findings of our study indicate that the SsRapid test demonstrates superior diagnostic performance compared to the in-house NIE-ELISA and offers notably higher sensitivity than the parasitological method when evaluating a single stool sample from each participant. Additional research across diverse geographic regions and epidemiological settings is recommended to more accurately determine the specificity of the SsRapid test. Owing to its rapid format and user-friendly design, SsRapid presents a viable field-deployable option for integration into STH deworming initiatives focused on the serological detection of *S. stercoralis*.

## Supplementary Information

Additional file 1. Figure S1. Two-by-two tables comparing results of serological tests with copro-parasitological methods (sedimentation and Baermann) and the composite reference (copro-parasitology and NIE-ELISA). A: Comparison of the SsRapid and copro-parasitology methods. B: Comparison of the SsRapid with the composite reference. C: Comparison of the in-house NIE-ELISA and copro-parasitology methods.

Additional file 2. Table S1. Intestinal parasites were detected in single specimens from each participant (*n* = 327) of individuals living in indigenous villages in Puerto Iguazú, Misiones, Argentina.

Additional file 3. Dataset S1. Diagnostic results obtained from the three methods.

## Data Availability

Data supporting the main conclusions of this study are included in the manuscript.
