# Supplementary figures and images for: Diagnostic performance of a Strongyloides IgG4 Rapid Test in detecting human Strongyloides stercoralis infection
Source: Parasit Vectors. 2025 Dec 11;19:31. doi: 10.1186/s13071-025-07154-7 (PMC12802000; doi:10.1186/s13071-025-07154-7)

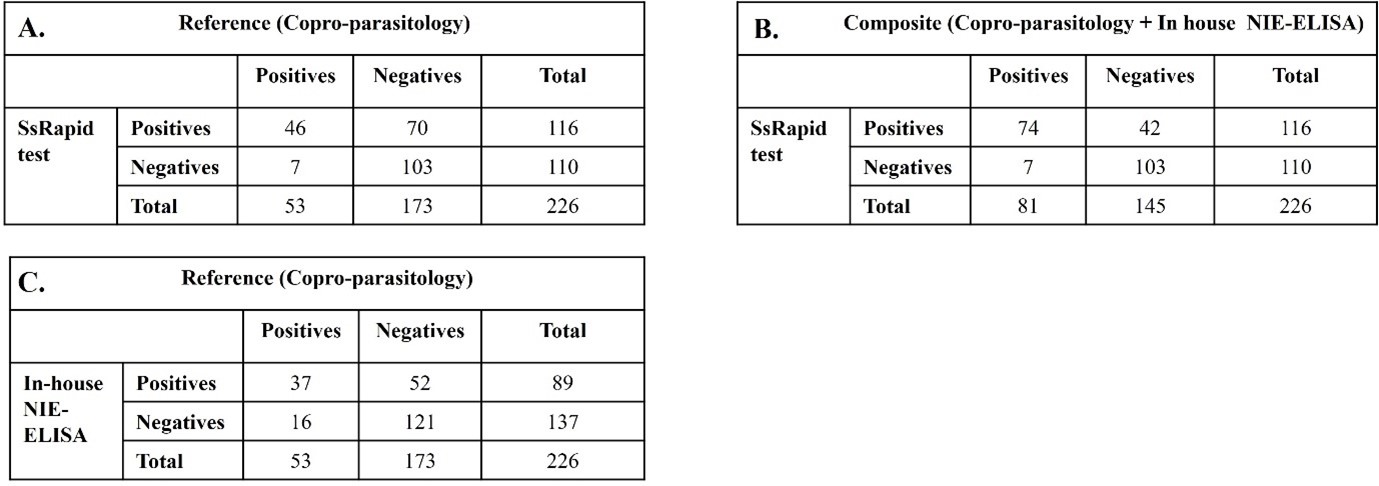

Supplement: Supplementary file 1 — Additional file 1. Figure S1. Two-by-two tables comparing results of serological tests with copro-parasitological methods (sedimentation and Baermann) and the composite reference (copro-parasitology and NIE-ELISA). A: Comparison of the SsRapid and copro-parasitology methods. B: Comparison of the SsRapid with the composite reference. C: Comparison of the in-house NIE-ELISA and copro-parasitology methods. [file 13071_2025_7154_MOESM1_ESM.jpg]
