# Supplementary material for: Diagnostic performance of a Strongyloides IgG4 Rapid Test in detecting human Strongyloides stercoralis infection
Source: Parasit Vectors. 2025 Dec 11;19:31. doi: 10.1186/s13071-025-07154-7 (PMC12802000; doi:10.1186/s13071-025-07154-7)
Supplement: Supplementary file 2 — Additional file 2. Table S1. Intestinal parasites were detected in single specimens from each participant (n = 327) of individuals living in indigenous villages in Puerto Iguazú, Misiones, Argentina. [file 13071_2025_7154_MOESM2_ESM.docx]

**Supplementary Information**

**Table S1**

Intestinal parasites were found in single specimens of each participant (n=327) from individuals living in indigenous villages in Puerto Iguazú, Misiones, Argentina.

| Parasites | Microorganisms | Samples | Number (N) | Prevalence (%) |
| --- | --- | --- | --- | --- |
| Hookworms | Soil-transmitted Helminths | 327 | 193 | 59.0 |
| *Strongyloides stercoralis* |  |  | 84 | 25.7 |
| *Ascaris lumbricoides* |  |  | 11 | 3.4 |
| *Trichuris trichiura* |  |  | 1 | 0.3 |
| *Blastocystis hominins* | Protozoa |  | 186 | 56.9 |
| *Entamoeba coli* |  |  | 161 | 49.2 |
| *Giardia lamblia* |  |  | 93 | 28.4 |
| *Entamoeba hartmanni* |  |  | 71 | 21.7 |
| *Hymenolepis nana* | Cestode |  | 67 | 20.5 |
| *Endolimax nana* | Protozoa |  | 58 | 17.7 |
| *Iodamoeba bütschlii* |  |  | 19 | 5.8 |
| *Chilomastix mesnili* |  | 288 | 15 | 5.2 |
| *Enterobius vermicularis* | Nematodes | 288 | 8 | 2.7 |
